# A patient with acute pulmonary embolism caused by hyaluronic acid injection underwent nursing care of extracorporeal membrane oxygenation support therapy: a case report

**DOI:** 10.3389/fmed.2025.1645839

**Published:** 2025-10-15

**Authors:** Yan Wang, Qidan Deng, Shili Tang, Fenhao Yu, Yunhao Lv, Zhi Liu, Lizhen Lin, Huihui Lu

**Affiliations:** ^1^Department of Intensive Care Unit, Affiliated Qingyuan Hospital, Guangzhou Medical University, Qingyuan People’s Hospital, Qingyuan, China; ^2^Department of Nursing, Affiliated Qingyuan Hospital, Guangzhou Medical University, Qingyuan People’s Hospital, Qingyuan, China; ^3^Department of Cardiovascular Medicine, Affiliated Qingyuan Hospital, Guangzhou Medical University, Qingyuan People’s Hospital, Qingyuan, China

**Keywords:** acute pulmonary embolism, extracorporeal membrane oxygenation, cardiac arrest, thrombolysis, nursing care

## Abstract

This article summarizes the nursing management of a patient who developed acute pulmonary embolism with concomitant cardiopulmonary arrest following intravaginal hyaluronic acid injection, requiring extracorporeal membrane oxygenation (ECMO) support. The main measures are rapid activation of the treatment plan to improve the efficiency of treatment; teamwork and safe transfer; early implementation of target temperature management to promote neurological prognosis; implementation of individualized anticoagulation strategies and infection control strategies; and autologous blood transfusion techniques to reduce blood loss during ECMO withdrawal. After 9 days of active treatment and refined care, the patient’s condition was stable, and she was transferred to the general ward to continue treatment for 2 days and was discharged after recovery. At 1-month follow-up after discharge, the patient’s consciousness was clear, her speech was clear, and the muscle strength of the limbs was back to normal. The cooperation of a mature ECMO team was important in the rescue and treatment of this patient, which could shorten the response time in all aspects of the rescue and improve the success rate of rescue and treatment. The application of individualized therapeutic measures and high-quality nursing care is the key to promote the recovery of this patient.

## 1 Introduction

Acute pulmonary thromboembolism (PTE) refers to a constellation of clinical and pathophysiological phenomena resulting from impaired respiratory and pulmonary circulatory functions. This condition arises due to the obstruction of pulmonary arterial branches or trunks caused by embolization of thrombi that originate predominantly in the deep veins of the lower extremities—a condition known as deep vein thrombosis (DVT)—and subsequently migrate to the pulmonary arteries (1, 2). In patients with acute pulmonary embolism, obstruction of the pulmonary arteries, reduction or even interruption of blood flow can trigger right ventricular failure, all of which are key contributors to the patient’s death (3). Among PTE patients, those who develop severe circulatory collapse within 30 days have a mortality rate of between 16 and 25%. In patients who develop complications of cardiac and respiratory arrest, the mortality rate is between 52 and 65%, and approximately 10% of patients die within the first hour of the onset of the disease (4, 5). Acute massive pulmonary thromboembolism (PTE) is a critical condition that can precipitate obstructive shock and cardiac arrest. It is distinguished by its rapid progression and high mortality rate if not promptly diagnosed and managed. The early use of veno-arterial extracorporeal membrane oxygenation (VA-ECMO) can significantly reduce the working pressure of the right ventricle and enhance the function of the right ventricle, thus ensuring hemodynamic stabilization and tissue oxygenation recovery (6). With the development of the medical cosmetic industry, complications after hyaluronic acid injection have gradually attracted attention, and pulmonary embolism, as one of the serious complications, poses a great threat to patients’ lives. The ICU plays a critical role in the management of such critically ill patients. Recently, the ICU of our hospital successfully employed VA-ECMO technology in the treatment of a patient who experienced cardiac arrest following a pulmonary thromboembolism (PTE) induced by an intravaginal hyaluronic acid injection. The following are some of our experiences and reports on emergency and nursing care.

## 2 Case presentation

We report the case of a 40-year-old Chinese female patient, was admitted to the hospital for “dizziness, chest tightness, and profuse sweating for more than 5 h.” The patient was admitted to our hospital at 20:30 on November 07, 2024, after an intravaginal injection of hyaluronic acid in a beauty institute, and the emergency personnel found that her blood pressure could not be measured at the scene, and she was admitted to our hospital after initial resuscitation. Emergency examination revealed significant enlargement of the right heart, suggesting shock secondary to right heart failure. Despite the administration of high-dose vasoactive agents, the patient’s blood pressure remained unstable, prompting the immediate initiation of ECMO rescue therapy. The emergency bedside ECMO procedure was performed at 00:35 on November 8. At 00:55, the patient’s heart rate decreased to 30 beats per minute, accompanied by loss of consciousness and absence of major arterial pulses. Resuscitative measures, including external chest compressions, endotracheal intubation for mechanical ventilation, and intravenous administration of epinephrine, were immediately instituted. Spontaneous heartbeat returned at 00:58, and ECMO major circulation was successfully established by 01:00.

When the patient was transferred to ICU, she had a temperature of 36.6 °C, heart rate of 113 beats/min, respirations of 12 beats/min (invasive ventilator-assisted ventilation), and a blood pressure of 85/74 mmHg (VA-ECMO maintenance circulation); she was in a state of psychiatric drug sedation and analgesia, with an acute facies, and cold and wet body. During the course of VA-ECMO, the patient was provided with a variety of therapies including target temperature management, sedation and analgesia, and anti-infection. Considering the patient’s sudden chest tightness, dizziness, and sweating, the patient performed vaginal hyaluronic acid injection and filling treatment in a beauty institution before the onset of the disease, and the injected material was crosslinked sodium hyaluronate gel, with an injection volume of 27 pcs (27 mL), which was combined with the emergency cardiac ultrasound of the right cardiac system significantly enlarged, pulmonary arterial hypertension, and the electrocardiogram suggesting the manifestation of SIQIIITIII, and although the lung CTA did not show any obvious changes, it still could not exclude that hyaluronic acid mistakenly enters into the vessels to cause particulate obstruction of the small branches of the pulmonary artery is possible, and the possibility of acute non-thrombotic pulmonary embolism and obstructive shock is currently considered to be high. Given the indeterminate etiology, a multidisciplinary consultation involving pulmonology, cardiology, pharmacy, aesthetic medicine, and other relevant specialties was convened to guide diagnosis and management. On the second day, the patient demonstrated improved neurological responsiveness, evidenced by the ability to withdraw the stimulated limb, prompting discontinuation of therapeutic hypothermia and initiation of controlled rewarming. Combined with the multidisciplinary joint consultation opinion agreed with the diagnosis of non-thrombotic pulmonary embolism, but due to the hyaluronic acid residue spread to the terminal branch vessels of the pulmonary artery, which was difficult to be dissolved by drugs, it was recommended to continue the VA-ECMO adjuvant therapy. Treatment was continued with anti-infection, maintenance of stable internal environment, nutritional support and other treatments, waiting for the recovery of cardiac function. On the fifth day, the patient’s consciousness was clear, the tidal volume was OK, and she could cooperate with the treatment, and she was given oxygen in the endotracheal tube, the patient’s spontaneous respiration was smooth, the depth of respiration was OK, respiratory rate was 17–20 times/min, cough reflex was good, and there was a small amount of white sputum in the endotracheal tube. The patient’s peripheral oxygen saturation was 99%, the respiratory sounds of both lungs were coarse, and a small amount of wet rales were detected in both lower lungs. Arterial blood gas analysis revealed the following: pH 7.47, whole blood base excess −0.9 mmol/L, extracellular fluid base excess −1.1 mmol/L, lactate 0.8 mmol/L, sodium 139 mmol/L, potassium 4.2 mmol/L, partial pressure of oxygen (PaO₂) 168 mmHg, partial pressure of carbon dioxide (PaCO₂) 31 mmHg. Considering that the patient’s consciousness was clear, cooperated with the treatment, and had good spontaneous respiration, she was given suction to clean the secretions in the oral cavity and tracheal tube, and the tracheal tube was extubated. On the sixth day, the patient’s heart rate increased to 135–140 beats/min during the VA-ECMO flow reduction test, and bedside ultrasound monitoring showed that the right heart was expanding and the left heart was contracting normally, considering that there was still the possibility of obstruction, and continued to give VA-ECMO assisted treatment. On the eighth day, the patient entered the programmed withdrawal, gradually down-regulated ECMO assisted flow, observed the heart rate, blood pressure to maintain stability, heart rate 80 beats/min, blood pressure about 125/55 mmHg, review of cardiac ultrasound patient’s cardiac contraction is better than before, aortic phase VTI more than 10 cm/s, then clamp the tube back to the 400 mL of blood clamped off the end of the arterial perfusion and the end of the venous blood diversion, respectively, to pull out the ECMO line, the puncture port localized deep purse-string suture, the ECMO line. On the ninth day, the patient’s condition was stable and she was transferred to the general ward for further treatment. On the eleventh day, the patient recovered well and was discharged.

## 3 Discussion

### 3.1 Rapid activation of ECMO treatment plan to improve treatment efficiency

Teamwork in extracorporeal cardiopulmonary resuscitation (ECPR) has been reported to not change the duration, hospitalization days, and mortality of ECMO, but it does improve the resuscitation outcome of ECPR (7). In 2015, an ECPR team was established in our hospital led by ICU 1, and more than 400 cases of ECMO have been performed so far. Emergency medical team doctors quickly judged that the patient met the indications for ECMO treatment, and immediately started the ECMO rapid response system, the ECMO team received the emergency call at 00:10, and the ECMO team of four health care workers arrived at the emergency resuscitation room at 00:30, doctor 1 talked to the family and signed the informed consent; doctor 2 prepared the skin, disinfection and other preparations; at the same time, nurse 1 inspected the operating environment, the meanwhile, nurse 1 inspected the operation environment, arranged the pre-filling position of the ECMO machine reasonably, connected the power supply, oxygen source and gas source, and prepared the pre-filling line; nurse 2 participated in the cooperation of tube placement, and passed the items in an orderly manner according to the operation procedure.

November 8, 00:35 Initiation of operation; Chen et al. (8) demonstrated that during cardiopulmonary resuscitation, circulatory perfusion decreases significantly over time, which leads to a lower success rate of vascular puncture, whereas percutaneous ultrasound guidance significantly improves the success rate of puncture. Expert consensus points out that the classic pathway for establishing VA-ECMO is the femoral vein (drainage cannula)—common femoral artery (perfusion cannula). The insertion site for the perfusion cannula can also be the subclavian artery or axillary artery. This pathway is used as an alternative when the femoral artery access is not feasible, but it may lead to arm swelling and excessive cerebral perfusion due to over-perfusion of the upper limbs. The carotid artery, when used as a perfusion cannula, is commonly chosen as the last option in adults due to the potential increased risk of acute brain injury and is not recommended (9). Therefore, after systemic heparinization of the patient, the team used ultrasound to assist in locating the puncture site and inserted a 21 Fr Maikewei ECMO venous drainage cannula along the guidewire, with a length of approximately 44 cm. Ultrasound confirmed that the tip of the drainage cannula was located at the opening of the right atrium. The procedure was continued with the same method for the right femoral artery puncture to place the arterial return cannula, inserting a 17 Fr Maikewei ECMO arterial return cannula along the guidewire to a depth of 15 cm. Wang et al. (10) found that the absence of distal perfusion catheters is an independent risk factor for acute limb ischemia. Preventive placement of distal perfusion catheters can reduce the occurrence of acute limb ischemia. To avoid distal limb ischemia in the right lower limb, selective right femoral artery puncture and catheter infusion were performed using the same method. A single-lumen 8 Fr venous catheter was inserted for distal perfusion of the right lower limb, approximately 15 cm in depth. Ultrasound confirmed that the catheter was located in the femoral artery. At 00:55 during ECMO cannulation, the patient experienced loss of consciousness and loss of aortic pulsation, ECPR was initiated rapidly, chest compressions, endotracheal intubation, ventilator-assisted ventilation, and epinephrine injection were performed immediately, and the patient’s heartbeat recovered at about 00:58, and the VA-ECMO was successfully switched to ECMO at 01:00. The whole operation process was 25 min.

### 3.2 Teamwork for safe transit

According to the guidelines and the “5P transfer system,” safe and rapid transfer was realized (11, 12). (1) Specialized ECMO personnel assess the condition and transfer route; during the transfer process, they are responsible for observing and adjusting the machine parameters and giving relevant medical advice; after the transfer, they work with specialized nurses to determine the machine position and sort out the ECMO tubing; (2) Prior to transport, the ECMO specialist nurse will meticulously inspect the function of all ECMO components, monitor the oxygen tank pressure, and organize and secure the ECMO circuits. Subsequently, they will collaborate with the attending physician to assess the patient’s condition and evaluate the efficacy of the nursing interventions; observe the ECMO tubing during the transfer process to prevent pulling and pulling; check the transfer items and medicines, comb the ventilator tubing and vascular access and fix them appropriately; and ensure the safety of the ventilator tubing and venous access during the transfer process; (3) The emergency department physician, based on the specialist’s assessment, explains the transport risks to the family, coordinates with the receiving unit, confirms the required diagnostic items, and issues corresponding medical orders prior to patient transfer. Under multidisciplinary collaboration, the medical and nursing team worked together to smoothly transfer the patient into ICU ward 1. The whole transfer process ensured that the patient’s vital signs were stable, the analgesic and sedative measures were appropriate, and the family highly recognized the efficiency of the transfer.

### 3.3 Early target temperature management favorable neurological prognosis

The American Heart Association (AHA) and the European Resuscitation Council (ERC) in their guidelines both recommend subcooling for cardiac arrest patients, taking various measures to lower the core body temperature of patients to achieve Targeted Temperature Management (TTM) to reduce the cerebral metabolic rate and reduce the degree of brain damage after cardiac arrest, and to improve survival and neurological prognosis (13, 14). The clinical value of TTM as a key intervention to improve the neurological prognosis of patients with cardiac arrest has been confirmed by several randomized controlled studies (15). After this patient underwent ECMO transfer, the temperature of the variable temperature water tank was rapidly adjusted to 35 °C, which rapidly brought the body temperature down to 35 °C and maintained it in this process for 24 h. After the end of the maintenance period, the patient entered the rewarming phase, which strictly followed the principle of stepwise warming, with a gradual recovery in the temperature range of 0.1–0.2 °C/h until the body temperature reached 36.5 °C, which was done in order to prevent rapid rewarming from leading to increased neurological damage. During the course of TIM treatment, the patient did not experience adverse symptoms such as chills or diarrhea. In this case, the patient underwent TTM therapy supported by extracorporeal membrane oxygenation (ECMO), which exemplifies the synergistic application of multimodal life support techniques in modern critical care medicine. The patient was followed up in the first month after discharge and was found to be still lucid, with a cerebral performance category (CPC) of 1 and a good neurological prognosis.

### 3.4 Anticoagulation management during ECMO operation

**Figure 1:**
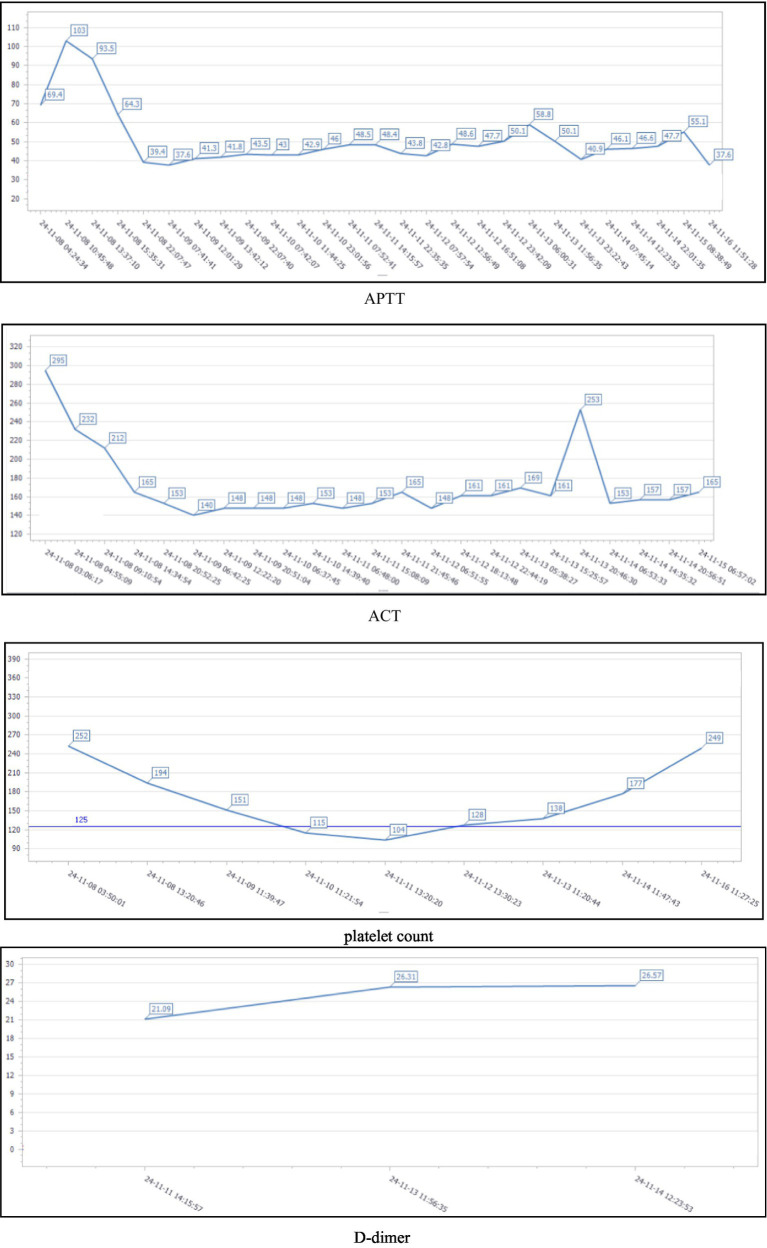
Dynamic changes in APTT, ACT, platelet count, and D-dimer during the patient’s hospitalization.

Systemic heparinized anticoagulation is critical to ensure that ECMO works properly (16, 17). Patients with ECMO frequently develop multiple complications, of which gastrointestinal bleeding and intracranial hemorrhage are two of the most serious, and IV thrombolysis, particularly when combined with the systemic anticoagulation required for ECMO support and especially in patients who have undergone recent CPR or surgical procedures, will inevitably increase the risk of major bleeding (18–20). The patient has a BMI of 17.1 and underwent prosthetic implantation in multiple sites, including the breast and hip, 3 years ago, with specific details unclear. The patient has no history of VTE. Upon admission, the initial ACT was 295 s, APTT was 69.4 s, and the platelet count was 252 × 10^9^/L. Therefore, a meticulous anticoagulation management plan was designed for the patient during the ECMO operation: During the operation phase of ECMO, we used sodium heparin at a dose of 5–40 IU/(kg/h) for continuous intravenous pumping and monitored the activated partial thromboplastin time (q4h) to ensure that the APTT was maintained in the range of 40–60 s and the ACT was maintained in the range of 180–220 s. At the same time, platelets, fibrinogen, and other indexes were dynamically rechecked (21). During the period of assistance, the patients’ APTT was up to 103 s, and ACT was maintained at 140–295 s. No obvious active bleeding was observed, and no complications such as thromboembolism occurred. Coagulation function was closely monitored during ECMO support, and specific values are shown in Figure 1. A moderate decrease in platelet count was noted post-ECMO initiation. Although heparin-induced thrombocytopenia (HIT) was a consideration, the clinical probability based on the 4 T’s score was low. The platelet count recovered without switching to an alternative anticoagulant like fondaparinux, suggesting a non-HIT etiology. Bedside ultrasound technology was used to monitor the patient’s cardiopulmonary function and the presence of pleural and abdominal fluid in real time to rule out thoracic and abdominal hemorrhage. On day 8, when the cardiac ultrasound examination confirmed that the patient’s cardiac function had improved, the patient’s heart rate and blood pressure performed well in the programmed withdrawal trial, and the ECMO was withdrawn in a timely manner.

### 3.5 Infection control strategies

Prevention and control of nosocomial infection during extracorporeal membrane oxygenation (ECMO) support is a critical aspect that affects patient prognosis. Evidence-based medical studies have shown that the incidence of nosocomial infections in ECMO patients fluctuates from 9 to 65%, which is significantly higher than that in the general population of critically ill patients, which is closely related to immunosuppression caused by extracorporeal circulation, multiple invasive operations, and prolonged exposure to the intensive care environment (22). In accordance with institutional protocol for all ECMO procedures, broad-spectrum antibiotic prophylaxis was administered following cannulation to mitigate the risk of nosocomial infections. In our center, we constructed an infection prevention and control system based on the multidisciplinary team (MDT) model, which consists of a core team of experts in critical care medicine, infection control, clinical pharmacy, microbiology and nursing, and we established a risk assessment matrix through the Delphi method, focusing on catheter-related bloodstream infection, CRBSI, and other infections. bloodstream infection (CRBSI), ventilator-associated pneumonia (VAP) and catheter-associated urinary tract infection (CAUTI). Precision prevention and control were implemented with the following specific intervention strategies:

#### 3.5.1 Catheter-related bloodstream infection prevention and control

In accordance with the guidelines of the American Society of Infectious Diseases, the “zero tolerance” vascular access management standard was established: chlorhexidine gluconate (2%) ethanol (70%) compound disinfectant was used to disinfect the vascular access three times in a spiral pattern (clockwise → counterclockwise → clockwise), the diameter of disinfection was >15 cm, and the drying time was strictly >2 min; “3-2-1” medication change mechanism was established: the first dressing change was performed 3 days after the puncture, and the dressing was changed every 2 days thereafter. The “3-2-1” dressing change mechanism: the first dressing change was performed 3 days after puncture, and the dressing was changed every 2 days thereafter, and blood/seepage >1 cm^2^ was found to be changed immediately, and the process of changing the dressing was strictly in accordance with the WHO five-moment principle of hand hygiene. Vascular ultrasound Doppler was applied to assess the catheter position and thrombosis risk weekly, and the anticoagulation regimen was adjusted in conjunction with the coagulation results.

#### 3.5.2 Prevention and control of ventilator-associated pneumonia

A modified version of the VAP intensive care strategy (Ventilator Bundle 2.0) was implemented: subglottic suction was performed using an intelligent negative pressure control system (−20 to −30 cmH_2_O), and pulsatile rinsing (5 mL of saline) combined with continuous low-negative-pressure suction (−5 cmH_2_O) was performed every 4 h. Oral care was performed by applying 0.12% chlorhexidine cotton balls for six-sided wiping method (buccal surface of teeth → tongue → occlusal surface → hard palate → buccal mucosa → sublingual), together with the electric toothbrush to mechanically remove the biofilm three times a day. The bed was maintained with the head of the bed elevated at 35 ± 5°, with real-time monitoring of angular excursions by means of a built-in accelerometer. The ventilator humidification tank used an active heating guide wire system to maintain the airway outlet gas temperature of 37 ± 0.5 °C and relative humidity of 100%, and the amount of condensate dumping was monitored every shift (>200 mL/day suggesting excessive humidification).

#### 3.5.3 Catheter-associated urinary tract infection prevention and control

Proper immobilization, keeping the urinary catheter airtight, drainage below the level of the bladder, timely dumping of urine, and perineal wiping twice a day. Perineal care was performed using a two-step method: first, the urethral opening to the perineum was scrubbed with 0.05% povidone-iodine cotton balls (unidirectional cleansing), and then rinsed and dried with saline.

#### 3.5.4 Protective isolation

Single room isolation, specialized care, no family visits, video visits. Pay attention to infection indicators such as white blood cells, C-reactive protein and calcitoninogen, as well as changes in body temperature.

With these interventions, no nosocomial infections occurred in this ECMO patient.

### 3.6 Autologous blood transfusion to reduce blood loss during ECMO withdrawal

Extracorporeal membrane pulmonary oxygenation (ECMO) support is often accompanied by significant blood component destruction and the need for allogeneic transfusion, and autologous blood reinfusion (ABR), an evidence-based hemoprotective strategy, has been demonstrated to reduce allogeneic red blood cell transfusion by up to 30 to 50%, as well as to reduce the risk of complications such as transfusion-related acute lung injury (TRALI) and the risk of complications such as transfusion-related circulatory overload (TACO) (23). Based on the International Extracorporeal Life Support Organization (ELSO) guidelines and the consensus on blood management, this study systematically describes the standardized operational procedures for autologous blood recovery during the withdrawal phase of ECMO.

Our center uses a modified closed blood recovery system to implement goal-oriented autologous blood recovery before ECMO withdrawal. The operation follows a “three-phase control method”:

Pre-flush phase: 0.9% saline (500 mL) was connected to the tee in front of the centrifugal pump, and the whole blood storage bag (500 mL) was connected to the side hole tee at the return end of the ECMO, the ECMO line was pre-flushed with 0.9% saline, and the centrifugal pump was connected to the centrifugal pump through the tee valve (with the rotational speed set to 2,500 ± 50 rpm) to maintain a pressure gradient in the line of less than 50 mmHg.

Blood collection stage: Clamp the blood-drawing end (venous side) and blood-returning end (arterial side) of the ECMO, open the three-way channel of the centrifugal pump and the channel of the blood-collection bag, introduce 0.9% NS into the ECMO line in negative-pressure suction mode (−100 to −150 mmHg), and the blood is injected into the blood-collection bag through the side hole of the arterial end, and the bag is gently shaken to make the blood and the preservative fluid mix well.

Blood retrieval phase: Within 30 min after withdrawal of the machine, blood was transfused at a rate of 2–4 mL/kg/h using a transfusion device treated with a transfusion warming device (37 ± 1 °C), with simultaneous monitoring of central venous pressure and mixed venous oxygen saturation to maintain hemodynamic stability. About 400 mL of autologous blood was eventually recovered, and the recovered blood was delivered to the patient according to the transfusion procedure after withdrawal of the machine.

## 4 Conclusion

Hyaluronic acid microparticles, often introduced into the systemic circulation through procedures such as cosmetic injections, may reach the lungs via blood flow if inadvertently entering vascular structures. Generally, larger microparticles (e.g., >100 μm) are more likely to occlude larger branches of the pulmonary arteries, while smaller particles (e.g., 10–100 μm) may travel into finer vasculature. When these microparticles lodge in pulmonary arteries or their branches-due to size compatibility with vascular diameters-they cause mechanical obstruction. As foreign entities, they trigger immune responses by attracting inflammatory cells. These cells release mediators such as tumor necrosis factor-alpha (TNF-*α*) and interleukin-6 (IL-6), inducing local inflammation, damaging vascular endothelial cells, and exacerbating tissue hypoxia and functional impairment. Concurrently, hyaluronic acid microparticles can disrupt the normal vasomotor function of pulmonary vessels, leading to increased pulmonary vascular resistance and exacerbating pulmonary hypertension. This impairs pulmonary gas exchange and, in severe cases, may result in critical outcomes such as respiratory failure. When hyaluronic acid is erroneously injected into vaginal blood vessels, it enters the circulatory system. As the lungs are a primary filtration organ, the circulating acid can form emboli that occlude the pulmonary arteries or their branches, causing pulmonary embolism. Furthermore, the injection procedure may induce local vascular injury and disrupt coagulation homeostasis, elevating the risk of thrombosis. These thrombi can subsequently dislodge and migrate to the pulmonary vasculature, causing embolic events. This case represents our institution’s first ECMO-treated patient with acute pulmonary embolism resulting from intravaginal hyaluronic acid injection, with no similar rescue experiences reported domestically or internationally. Multidisciplinary consultation agreed with the diagnosis of non-thrombotic pulmonary embolism, but due to the spread of hyaluronic acid residue to the terminal branches of the pulmonary artery, it was difficult to be dissolved by drugs, and there was no indication for interventional thrombolysis, so it was recommended to continue VA-ECMO-assisted treatment, and the patient was discharged from the hospital after active resuscitation and nursing care. The professional ECMO team can quickly start the ECMO treatment plan, early management of target body temperature can promote the prognosis of neurological function, personalized anticoagulation method and autologous blood transfusion technology to prevent bleeding in patients with high bleeding risk and improve the efficiency of treatment. This case provides a novel therapeutic direction and offers valuable insights for the management of acute pulmonary embolism resulting from intravaginal hyaluronic acid injections.

## Data Availability

The original contributions presented in the study are included in the article/supplementary material, further inquiries can be directed to the corresponding author.
